# Prognostic impact of H3K27me3 expression on locoregional progression after chemoradiotherapy in esophageal squamous cell carcinoma

**DOI:** 10.1186/1471-2407-9-461

**Published:** 2009-12-22

**Authors:** Li-Ru He, Meng-Zhong Liu, Bin-Kui Li, Hui-Lan Rao, Yi-Ji Liao, Xin-Yuan Guan, Yi-Xin Zeng, Dan Xie

**Affiliations:** 1 State Key Laboratory of Oncology in South China, Cancer Center, Sun Yat-Sen University, No 651, Dongfeng Road East, 510060 Guangzhou, China; 2 Department of Radiotherapy, Cancer Center, Sun Yat-Sen University, No 651, Dongfeng Road East, 510060 Guangzhou, China

## Abstract

**Background:**

Trimethylation of lysine 27 on histone H3 (H3K27me3) by enhancer of zeste homolog 2 (EZH2) is an epigenetic mark that mediates gene silencing. EZH2 is overexpressed and correlates with poor prognosis in many cancers. However, the clinical implication of H3K27me3 in human malignancies has not been well established. We wished to ascertain whether a correlation exists between the expression of H3K27me3 and clinical outcome in a group of patients with esophageal squamous cell carcinoma (ESCC) treated with definitive chemoradiotherapy (CRT).

**Methods:**

The method of immunohistochemistry (IHC) was utilized to examine the protein expression of H3K27me3 in 98 pretreatment biopsy specimens of ESCC and in 30 samples of normal esophageal mucosa. The clinical/prognostic significance of H3K27me3 expression was statistically analyzed.

**Results:**

The expression frequency and expression levels of H3K27me3 were significantly higher in ESCCs than in normal tissues. There was a positive correlation between H3K27me3 expression and WHO grade (*P *= 0.016), tumor size (*P *= 0.019), T status (*P *= 0.024), locoregional progression (*P *= 0.009) and EZH2 expression (*P *= 0.036). High H3K27me3 expression was associated with poor locoregional progression-free survival (LPFS) (*P *= 0.010) in ESCC. Further analysis demonstrated that H3K27me3 could stratify patient outcome in T2-3 (*P *= 0.048), N0 (*P *= 0.005) and M0 (*P *= 0.018) stages as well as in CRT effective group (*P *= 0.022).

**Conclusions:**

Our data suggests that H3K27me3 expression examined by IHC might be useful for stratifying LPFS for different subsets of ESCC patients treated with definitive CRT.

## Background

Esophageal squamous cell carcinoma (ESCC) is an aggressive human cancer with poor prognosis worldwide [1]. Most patients present with locally advanced disease, and definitive chemoradiotherapy (CRT) is an important component of the therapeutic strategy for ESCC [2]. Despite the great advances achieved in radiotherapy technology recently, its overall 5-year survival rate remains less than 30%, and the high probability of recurrence is still the main cause of poor quality of life and death [3]. At present, only the stage based on Tumor Node Metastases (TNM) classification and primary complete response to CRT are widely accepted as prognostic factors [4]. However, there are substantial differences in survival within patients with the same clinical stage and/or CRT response, probably attributable to the differences in biologic behavior of the tumors. Improved prognostic markers that can further stratify patient outcome are therefore needed.

It is known that epigenetic changes, including DNA methylation and covalent histone modification, are involved in tumorigenesis and tumor progression of human cancers [5,6]. One of the most important mechanisms is that many cancer-related genes are silenced by the enhancer of zeste homolog 2 (EZH2), which can specially trimethylate lysine 27 on histone H3 (H3K27) of the target gene promoters [7]. EZH2 is overexpressed and correlates with poor prognosis in many cancers [8-13], however, the status of H3K27 methylation and its clinical implication in cancer patients are rarely studied. To date, the role of trimethylated H3K27 (H3K27me3) expression in patient outcome for different types of human cancer is still elusive [8,14,15], further investigations in different cohorts of cancer patients are actually needed. Thus, we performed the present study to investigate the clinical/prognostic implication of H3K27me3 in ESCC patients treated with definitive CRT.

## Methods

### Patients and tissue specimens

Ninety-eight primary ESCC patients treated with definitive CRT were consecutively selected from the Department of Radiotherapy, Cancer Center, Sun Yat-Sen University between January 2002 and December 2008. Tumor grade and stage were defined according to the 6^th ^edition of the TNM classification of the International Union Against Cancer (UICC, 2002). Clinicopathologic characteristics are summarized in Table 1. Patients with distant metastases except for supraclavicular or celiac lymph nodes were excluded from this study. All the patients received the same PF regimen (Cisplatin 80 mg/m^2 ^i.v. drip day 1, 28; 5-fluorouracil 3 g/m^2 ^c.i.v. day 1 to 2, 28 to 29) concurrently with radiotherapy (60-70 Gy, 1.8-2 Gy/fraction, 5 days a week). The tumor biopsy specimens were recruited from paraffin blocks of the 98 primary ESCCs from the Department of Pathology of our institutes. In addition, 30 biopsy samples of normal esophageal mucosa from the same patients from regions that were not affected were used for controls. The study was approved by the medical ethics committee of our institute.

**Table 1:** H3K27me3 expression and clinicopathologic variables (Chi-square test)

		H3K27me3 expression (%)		
		
Variables	Case	low	high	***P***
Age (years)				0.255
≤55^a^	54	32(59.3)	22(40.7)	
>55	44	21(47.7)	23(52.3)	
Gender				0.720
Male	82	45(54.9)	37(45.1)	
Female	16	8(50.0)	8(50.0)	
Location				0.341
Cervical	24	15(62.5)	9(37.5)	
Thoracic	74	38(51.4)	36(48.6)	
WHO grade				0.016
G1	24	16(66.7)	8(33.3)	
G2	50	30(60.0)	20(40.0)	
G3-4	24	7(29.2)	17(70.8)	
Tumor size (cm)				0.019
≤6^b^	56	36(64.3)	20(35.7)	
>6	42	17(40.5)	25(59.5)	
T status				0.024
T2-3	47	31(66.0)	16(34.0)	
T4	51	22(43.1)	29(56.9)	
N status				0.929
N0	16	8(50.0)	8(50.0)	
N1	82	40(48.8)	42(51.2)	
M status				0.651
M0	59	33(55.9)	26(44.1)	
M1-lym^c^	39	20(51.3)	19(48.7)	
CRT response				0.094
Effective	61	37(60.7)	24(39.3)	
Resistant	37	16(43.2)	21(56.8)	
Locoregional progression				0.009
Absent	51	34(66.7)	17(33.3)	
Present	47	19(40.4)	28(59.6)	
Distant progression				0.299
Absent	62	36(58.1)	26(41.9)	
Present	36	17(47.2)	19(52.8)	

^a^Mean age. ^b^Mean tumor size. ^c^Distant lymph node metastasis.

### Evaluation and follow-up

The effect of CRT was evaluated clinically for primary lesions based on esophagography and computed tomography (CT) 4 weeks after CRT according to World Health Organization (WHO) criteria. Complete response (CR), partial response (PR), no change (NC), and progressive disease (PD) were achieved in 19 patients, 42 patients, 36 patients and 1 patient, respectively. Thus, 61 cases were included in the effective group (CR/PR), the remaining 37 cases were included in the resistant group (NC/PD).

The patients were followed every 3 month for the first year and then every 6 months for the next 2 years, and finally annually. Of the 79 patients who didn't get CR, 22 cases received adjuvant chemotherapy, 2 cases received radical esophagectomy. The other patients didn't receive any anti-tumor treatments until tumor progression. The diagnostic examinations consisted of esophagography, CT, chest x-ray, abdominal ultrasonography and bone scan when necessary to detect recurrence and/or metastasis. Locoregional progression was defined as cases in which the primary tumor and regional enlarged lymph nodes evaluated as PD after CRT or recurrence after CR. Distant progression was defined as a failure control of the distant metastatic lymph nodes and/or a new distant metastasis occurred.

### Immunohistochemistry (IHC)

**Figure 1:**
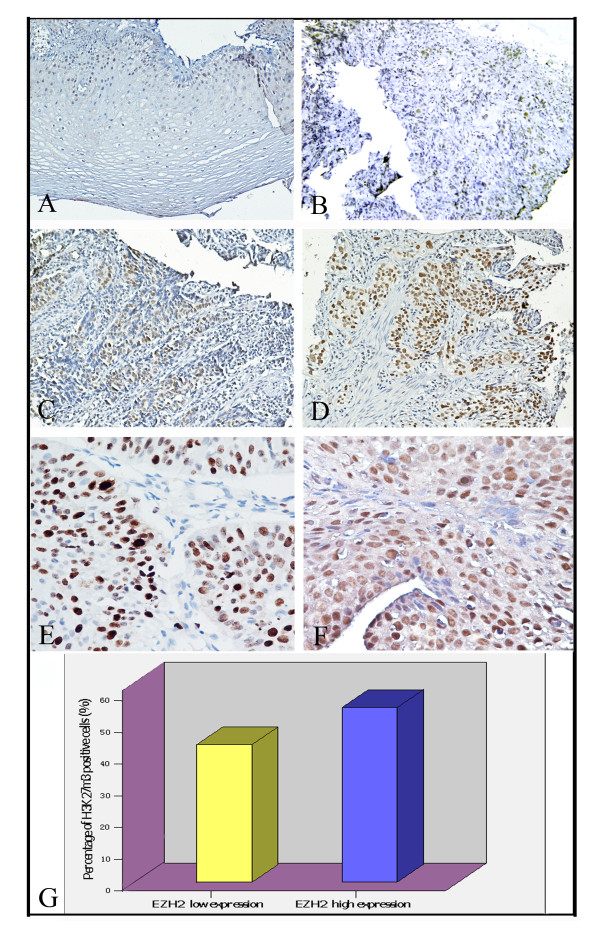
**Immunohistochemical stainings of H3K27me3 in human esophageal tissues**. (A) A normal esophageal mucosa (case 13) showed low expression of H3K27me3 protein, in which less than 50% of normal esophageal squamous cells showed positive staining of H3K27me3 in nuclei (200×). (B) An ESCC case (case 21) demonstrated low expression of H3K27me3, in which less than 50% of squamous cell carcinoma cells showed positive staining of H3K27me3 protein in nuclei (200×). (C) High expression of H3K27me3 was observed in an ESCC (case 39), where more than 70% carcinoma cells demonstrated positive staining of H3K27me3 in nuclei (200×). (D) A primary ESCC (case 52) was observed high expression of H3K27me3, where more than 90% carcinoma cells showed positive staining of H3K27me3 protein in nuclei (200×). (E) Another ESCC (case 7) was observed high expression of EZH2, where more than 90% of carcinoma cells showed positive staining of EZH2 protein in nuclei (400×). (F) High expression of H3K27me3 (more than 80% of carcinoma cells showed positive expression of H3K27me3) was observed in the same ESCC case 7 (400×). (G) For the 53 ESCC cases with high EZH2 expression, an average of 55.0% of the ESCC cells stained positive with H3K27me3 antibody, a percentage of cancer cells that was significantly larger than that (43.3%) in the remaining 45 cancers with a low expression of EZH2 (*P *= 0.036, independent sample t test).

IHC staining was performed on 5-μm tissue sections rehydrated through graded alcohols. Endogenous peroxidase activity was blocked with 0.3% hydrogen peroxide for 15 min. For antigen retrieval, tissue slides were boiled in 10 mM citrate buffer (pH 6.0) and microwave-treated for 10 min (H3K27me3), or in tris (hydroxymethyl) aminomethane-EDTA buffer (pH 8.0) in a pressure cooker for 12 min (EZH2). Nonspecific binding was blocked with 10% normal rabbit serum for 20 min. The tissue slides were incubated with anti-H3K27me3 (Abcam, Cambridge, MA, 1:50 dilution) or anti-EZH2 (BD Transduction Laboratories, Franklin Lakes, NJ, 1:100 dilution) for 60 min at 37°C in a moist chamber. Subsequently, the slides were sequentially incubated with biotinylated rabbit antimouse immunoglobulin at a concentration of 1:100 for 30 min at 37°C and then reacted with a streptavidin-peroxidase conjugate for 30 min at 37°C and 3'-3' diaminobenzidine as a chromogen substrate. The nucleus was counterstained using Meyer's hematoxylin. A negative control was obtained by replacing the primary antibody with a normal murine IgG. Positive expression of H3K27me3 and EZH2 in ESCC and normal esophageal mucosa cells was primarily a nuclear pattern (Fig. 1). Known immunostaining positive slides were used as positive controls.

Two independent observers blinded to the clinicopathologic information performed the scoring of H3K27me3 [14,15] and EZH2 [16-18] expression using their previously validated scoring systems respectively. For H3K27me3, the system calculated the percentage of nuclei staining positive for the H3K27me3 protein as close as to the multiples of 10. Since the frequency for the percentage of positively stained cells in all tumor samples assessed for H3K27me3 was almost a normal distribution with a range from 0% to 100%, and the median value was 50%, thus, categories of high and low expression were defined as groups with percentage of positive cells above or below/equal to 50% [14,15]. For EZH2, the system scores nuclear EZH2 expression by recording the percentage of nuclei staining positive for the EZH2 protein, in which EZH2 immunoreactivity was classified into two groups: low expression, when positive cells were less than 50%; and high expression, when at least 50% of the cells showed positive immunoreactivity in the nuclei [16-18]. In this study, a minimum of 500 epithelial cells was counted for each normal or tumor case.

### Statistical analysis

Statistical analysis was performed with the SPSS software (SPSS Standard version 13.0, SPSS Inc.). The association of H3K27me3 expression with ESCC patient's clinicopathologic features was assessed by the Chi-square test. Locoregional progression-free survival (LPFS) was defined as the time from diagnosis to tumor locoregional progression (the first end-point) or cancer-relative death (the second end-point). Disease-specific survival (DSS) was defined as the time from diagnosis to cancer-relative death. LPFS and DDS were assessed with the Kaplan-Meier method and compared by the log rank test. Multivariate survival analysis was performed on all the parameters that were found to be significant on univariate analysis using the Cox regression model. An independent sample t test was used to assess the expression of H3K27me3 between groups with low and high expression of EZH2. *P *values of < 0.05 were considered significant.

## Results

### Expression of H3K27me3 in ESCC

In the present study, protein expression of H3K27me3 was examined by IHC in 98 cases of primary ESCC and in 30 cases of normal esophageal mucosa. H3K27me3 was detected in 85.7% (84/98) of ESCCs, whereas in normal esophageal tissues the expression of H3K27me3 (19/30) was significantly lower (63.3%, *P *= 0.007). Using the criteria described above, high expression of H3K27me3 was observed in 45.9% (45/98) of the ESCCs and only 20.0% (6/30) of the normal esophageal tissues (*P *= 0.011). The association between clinicopathologic features and H3K27me3 expression levels of the 98 ESCCs were summarized in Table 1. The expression of H3K27me3 correlated closely with WHO grade (*P *= 0.016), tumor size (*P *= 0.019) and T status (*P *= 0.024). No significant association was found between CRT response and H3K27me3 expression (*P *= 0.094) or any clinicopathologic variables, such as patient's age, gender and tumor grade, location, size, T status and radiotherapy dose (60GY *vs*. > 60GY) (*P *> 0.05).

### Correlation between clinicopathologic variables, H3K27me3 expression and ESCC patient survival

Of the 98 ESCC patients, none was lost to follow-up. The median observation period was 23.9 months (2.3-80.7 months), with 47 tumor locoregional progressions, 36 distant progressions and 68 cancer-related deaths. The 5-year LPFS and DSS for the entire cohort of patients were 18.5% and 22.1%, respectively.

**Figure 2:**
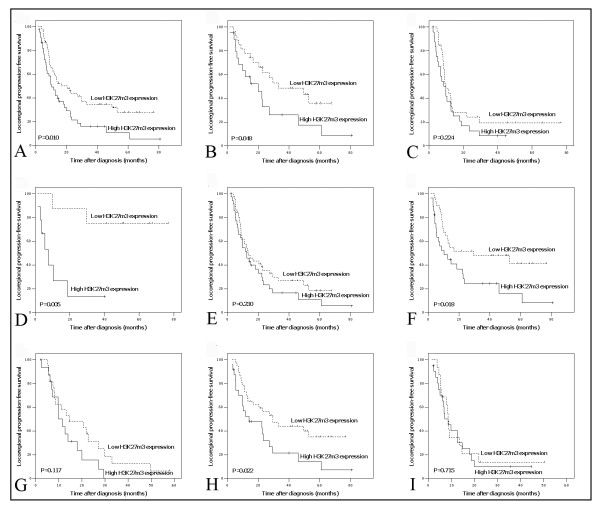
**Locoregional progression-free survival curves for total cohort and different subsets of ESCC patients**. (A) Total patients, (B) T2-3 subset, (C) T4 subset, (D) N0 subset, (E) N1 subset, (F) M0 subset, (G) M1-lym subset, (H) Chemoradiotherapy effective and (I) chemoradiotherapy resistant subsets: low expression (dashed line), high expression (solid line).

A positive association between high expression of H3K27me3 and the present of locoregional progression was demonstrated by our Chi-square test (Table 1). In univariate analysis, although there was no significant difference in DSS between groups with high and low expression of H3K27me3 (median 20.0 *vs*. 22.6 months, *P *= 0.151), high H3K27me3 expression was evaluated to correlate closely with poor LPFS (*P *= 0.010) for the ESCC patients (Fig. 2, Table 2). In stratified survival analysis, H3K27me3 expression could differentiate LPFS of the patients in T2-3, N0 and M0 stages, as well as in CRT effective group (Fig. 2, Table 2), however, no stratified significance of H3K27me3 expression was observed in DSS (*P *> 0.05).

**Table 2:** Univariate analysis of H3K27me3 for LPFS (log-rank test)

		LPFS (months)		
		
Variables	Case	Mean	Median	*P*-value
Total				0.010
Low expression	53	33.5	20.6	
High expression	45	19.4	10.4	
T status				
T2-3				0.048
Low expression	31	39.4	33.1	
High expression	16	25.9	20.0	
T4				0.224
Low expression	22	23.6	9.7	
High expression	29	12.9	8.6	
N status				
N0				0.005
Low expression	8	62.5	NR^a^	
High expression	8	11.9	7.4	
N1				0.230
Low expression	40	26.2	13.6	
High expression	42	20.5	12.7	
M status				
M0				0.018
Low expression	33	40.8	29.5	
High expression	26	22.6	10.4	
M1-lym				0.117
Low expression	20	20.5	14.7	
High expression	19	13.4	9.9	
CRT response				
Effective				0.022
Low expression	37	40.2	29.6	
High expression	24	24.0	14.1	
Resistant				0.715
Low expression	16	14.9	8.6	
High expression	21	12.6	8.5	

^a ^Not reached.

Kaplan-Meier analysis also demonstrated a significant impact of certain clinicopathologic prognostic parameters such as T status (*P *= 0.002), CRT response (*P *0.001) and M-lym status (*P *= 0.043) on LPFS. No significant association was found between survival and other clinicopathologic variables, including radiotherapy dose and receiving adjuvant chemotherapy or not (*P *> 0.05). The parameters that were significant in univariate analysis were further examined in multivariate analysis. The results showed that T status, CRT response and M-lym status were independent predictors of LPFS, while H3K27me3 expression only showed a borderline significance (*P *= 0.065, Table 3).

**Table 3:** Multivariate Cox regression analysis for LPFS

	LPFS	
	
Variable	HR^a^(95% CI^b^)	*P*
H3K27me3 expression		0.065
Low	1.000	
High	1.568 (0.972-2.528)	
CRT response		0.023
Effective	1.000	
Resistant	1.820 (1.084-3.055)	
T status		0.013
T2-3	1.000	
T4	1.376 (1.068-1.772)	
M status		0.029
M0	1.000	
M1-lym	1.721 (1.056-2.806)	

^a^Hazard ratio. ^b^Confidence interval.

### Correlation between the expression of H3K27me3 and EZH2 in ESCCs

Using the criteria described above, high expression of EZH2 was observed in 53/98 (54.1%) of the ESCCs (Fig. 1F) , the other 45 cases showed low expression of EZH2 (datas in submission). Thus, we further evaluated the relationship between the expression of H3K27me3 and EZH2. The results showed a positive correlation between the expression levels of H3K27me3 and EZH2. In the 53 ESCC cases with high EZH2 expression, an average of 55.0% of the ESCC cells stained positively with H3K27me3 antibody, a percentage of cancer cells that was significantly larger than that (43.3%) in the remaining 45 cancers with a low expression of EZH2 (*P *= 0.036, Fig. 1G).

## Discussion

Since ESCC patients with the same TNM stage and/or CRT response often display considerable variability in disease progression and survival, the traditional grading system may have reached its limits in providing critical information influencing patient prognosis and treatment strategies. Thus, novel diagnostic and risk assessment are urgently needed. Recently, histone modification, which is important for normal cell growth, has become an increasingly important aspect of cancer biology [6,19-21]. One such modification, the trimethylation of lysine 27 on histone H3 (H3K27me3) is required for Polycomb Repressive Complex 2 (PRC2) mediated repression of a large number of genes essential for cell proliferation, cell differentiation and tumor development [22,23]. It is believed that maintenance of the H3K27me3 mark during cell division is essential for normal embryogenesis and for preserving cell identity [24,25]. In human cancer, H3K27me3 has been evaluated as a prognostic factor in prostate, breast, ovarian, pancreatic and esophageal cancers [8,14,15], however, some of the results are totally contradictory. In the present study, we examined the expression of H3K27me3, in 30 samples of normal esophageal mucosa and 98 biopsy specimens of primary ESCCs treated with definitive CRT.

Our results showed that a significant percentage of the normal esophageal mucosa was observed positive staining of H3K27me3. However, the frequency of positive expression and levels of H3K27me3 in our ESCC cohorts were significant higher than that in normal esophageal tissues. Further analysis demonstrated that high expression of H3K27me3 was associated closely with poor tumor differentiation and advanced local invasion in ESCC, which was in general agreement with findings revealed by Ching et al [15]. In addition, we did further found that H3K27me3 expression correlated significantly with tumor locoregional progression rather than distant metastasis. These findings provide evidence that the up-regulation of H3K27me3 may provide a selective advantage for carcinogenesis and tumor progression of ESCC.

When focus on locoregional progression-free survival (LPFS) of ESCC, we found that high expression of H3K27me3 correlated with poor prognosis. Furthermore, the difference in H3K27me3 expression levels was evaluated to be a better predictor of LPFS when patients are stratified on the basis of T, N, M status and CRT response. It has been revealed that H3K27 methylation was mediated by the primary H3K27 methyltransferase, EZH2, a component of the Polycomb (PcG) complex that is involved in early carcinogenesis [26]. As aforementioned, our results showed that H3K27me3 predicted LPFS predominately in T2-3, N0 and M0 stages of ESCC may partly support the link between PcG complex and H3K27me3 in early carcinogenesis. Similar results were also reported in surgical treated ESCC patients by Ching et al [15]. In our CRT treated ESCC cohort, we further demonstrated that H3K27me3 could better stratify LPFS for CRT effective patients. In addition, ESCC patients with high expression of H3K27me3 were more likely to present with large tumor size and advanced T status; such may be at higher risk to suffer from tumor progression after CRT. Thus, our findings might be helpful to better understand the heterogeneity in the prognosis of ESCC patients within the same stages and even with a good response to CRT. Considering the potentially important role of H3K27me3 as a biological mechanism in locoregional progression of ESCC, the examination of H3K27me3 expression by IHC, therefore, might be used as an additional tool in risk assessment and therapy optimizing.

With regards to the biologic function of H3K27me3, it has been observed that, H3K27me3 was linked to PcG-mediated suppression of *homeotic box (Hox) *genes and maintenance of embryonic stem cell identity [27], whereas during embryogenesis and stem cell differentiation, consistent with the strong decrease in H3K27me3 levels associated with *HOX *genes expression, the level of EZH2 also declined [28,29]. Our results supported the role of H3K27me3 in cell differentiation of ESCC. Since methylation of H3K27 mediated by EZH2 has been implicated in the aggressive phenotype of cancer cells through repression of a panel of tumor suppression genes [30,31], the loss of function of these genes, in turn, locks stem/precursor cells into abnormal clonal expansion which begins a process of neoplastic initiation [32,33]. This might provide a possible explanation for the association of H3K27me3 expression and local invasion in ESCC found by our study. Moreover, an imbalance of H3K27 methylation owing to overexpression of EZH2 has been implicated in metastatic prostate and aggressive breast cancers [8,9], in which a highly significant overlap between PRC2- and H3K27me3-occupied genes was observed [8]. To determine whether there was a potential correlation between the expression of EZH2 and H3K27me3 in ESCC, we evaluated the expression status of the two proteins by IHC in the same cohort of cases. Our results demonstrated that the expression level of H3K27me3 in high EZH2 expression group was significantly higher than that in low EZH2 expression group, which supported the view that up-regulated expression of H3K27me3 in ESCCs might be caused, at least partly, by increased expression of EZH2. In other types of human cancer, in contrast, Wei et al reported that loss of H3K27me3 correlated with poor prognosis of patients with breast, ovarian, and pancreatic cancers, in which no association was observed between expression of H3K27me3 and EZH2 [14]. Considering that the mechanism by which EZH2-mediated H3K27 methylation leads to gene silencing may vary among gene targets and among organisms [34], discrepancies in different studies may arise. In addition, recent studies demonstrate that the H3K27me3 mark is more dynamic than previously anticipated and suggest that the levels of H3K27me3 is fine-tuned by opposing activities: demethylases and the PRC2 complex [27,35]. These results, collectively, suggested that the regulation of H3K27me3 expression is quite complicated and its biologic/clinical significance in different human cancers may be tissue-specific. Clearly, further investigations are substantially needed.

## Conclusion

In summary, in this study, we describe protein expression of H3K27me3 and its correlation with EZH2 expression in normal human esophageal tissues and in ESCCs. Our results provided, for the first time, a basis for the concept that high expression of H3K27me3, as detected by IHC, might be useful for stratifying LPFS for different subsets of ESCC patients treated with definitive CRT.

## Competing interests

The authors declare that they have no competing interests.

## Authors' contributions

LRH evaluated the clinical records, carried out the immunohistochemistry assays and drafted the manuscript. MZL designed the study and participated in its coordination. BKL participated in the statistical analysis and help to draft the manuscript. HLR performed the immunohistochemical analyses. YJL help to carry out the immunohistochemistry assays. XYG, YXZ and DX participated in the design of the study, in its analysis and in the interpretation of the data. DX also participated in evaluated the immunohistochemistry results and wrote the manuscript. All authors read and approved the final manuscript.

## Pre-publication history

The pre-publication history for this paper can be accessed here:

http://www.biomedcentral.com/1471-2407/9/461/prepub
